# Seroprevalence and Risk Factors Associated with Phleboviruses and Crimean–Congo Hemorrhagic Fever Virus among Blood Donors in Central Tunisia

**DOI:** 10.3390/pathogens13040348

**Published:** 2024-04-22

**Authors:** Rym Ayari, Houda Chaouch, Stephen Findlay-Wilson, Wissem Hachfi, Nadia Ben Lasfar, Foued Bellazreg, Stuart Dowall, Neila Hannachi, Amel Letaief

**Affiliations:** 1 Infectious Diseases Department, Farhat Hached University Hospital, Sousse 4000, Tunisia; rym_ayari@yahoo.com (R.A.); chawech_houda@yahoo.fr (H.C.); wissemhachfi@gmail.com (W.H.); benlasfar.nadia@yahoo.fr (N.B.L.); fouedbellazreg@yahoo.fr (F.B.); 2 UK Health Security Agency (UKHSA), Porton Down, Salisbury SP4 0JG, UK; stephen.findlay-wilson@ukhsa.gov.uk (S.F.-W.); stuart.dowall@ukhsa.gov.uk (S.D.); 3 Faculty of Medicine, University of Sousse, Sousse 4000, Tunisia; naila.hannachi@rns.tn; 4 Microbiology Laboratory, Farhat Hached University Hospital, Sousse 4000, Tunisia

**Keywords:** seroprevalence, arboviruses, blood donors, phleboviruses, surveillance

## Abstract

The aim of this study was to determine the prevalence of six viruses, from two families of the order *Bunyavirales*, in the general population of central Tunisia. Sera collected from 377 asymptomatic blood donors were serologically assayed for Rift Valley fever virus (RVFV), Crimean–Congo hemorrhagic fever virus (CCHFV), and four sandfly-borne phleboviruses: Toscana virus (TOSV), sandfly fever Naples virus (SFNV), sandfly fever Sicilian virus (SFSV), and sandfly fever Cyprus virus (SFCV). Of the 377 subjects enrolled in this study, 17.3% were IgG positive for at least one of the viruses tested. The most frequently detected antibodies were against TOSV (13.3%), followed by SFCV (2.9%), RVFV (1.9%), SFSV (1.3%), and SFNV (1.1%). Only one sample was IgG positive for CCHFV. Dual reactivity was observed in nine cases: SFSV + SFCV in three cases (0.8%) and TOSV + SFNV, TOSV + SFCV, and TOSV + RVFV in two cases (0.5%) each. 15.9% of donors were IgG positive against sandfly-borne phleboviruses. Among the 65 donors IgG positive for phleboviruses, 50.8% were from rural areas compared to 12.3% from urban areas (*p* < 0.001); 92.3% had animals in their living quarters (*p* = 0.009); and 70.8% lived in the vicinity of stagnant water (*p* = 0.062). Seroprevalence was significantly higher among donors living with chronic diseases (*p* = 0.039). Furthermore, the seroprevalence of phleboviruses was higher in Kairouan, the central governorate, than in the two coastal governorates: Monastir and Sousse, with 33.4%, 24.2%, and 14.9%, respectively. The presence of antibodies in the general population needs further investigation to better assess the extent of these viruses. Only TOSV was known to have an extensive circulation in Tunisia and in North Africa. Continued surveillance and interventions are necessary to detect the emergence of all arboviruses and to prevent further transmission.

## 1. Introduction

The last few decades have been characterized by the global emergence of many new infectious diseases in the human population caused by arthropod-borne viruses (arboviruses) which have animal reservoirs [1,2]. Human populations in extensive geographical areas (southern Europe, Africa, the Middle East, central and western Asia) are at risk of infection with arboviruses causing hemorrhagic and neurologic diseases due to the presence of vectors [3]. The genetic plasticity of many viruses increases their ability to adapt to new environments and can lead to their emergence in new territories [1].

In countries bordering the Mediterranean basin, mosquitoes and ticks are involved in the transmission of several arboviruses [4]. These vectors play an important role in the introduction of arboviruses to human populations [5]. While the majority of human infections are asymptomatic, there are several arboviruses that can cause diseases of the central nervous system in vertebrate hosts [6]. Various species of virus from the order *Bunyavirales* occur in the Mediterranean region and cause neuroinvasive and hemorrhagic diseases, including viruses from two different families: *Phenuiviridae* and *Nairoviridae*. The most frequently detected viruses include: Rift Valley fever virus (RVFV), Crimean–Congo hemorrhagic fever virus (CCHFV), and members from the sandfly-borne phleboviruses (Toscana virus, TOSV; sandfly fever Naples virus, SFNV; sandfly fever Sicilian virus, SFSV; and sandfly fever Cyprus virus, SFCV) [7,8].

RVFV, typically transmitted by the bite of an infected mosquito from the *Aedes* genus [9], is endemic in sub-Saharan Africa and Egypt, where epidemics have great consequences for human health [10,11]. RVFV can cause serious complications including meningoencephalitis and hemorrhagic fever [12]. It is considered a serious emerging threat to public health and food security [13]. Its circulation has mostly been reported in domestic and wild animals [14].

The sandfly-borne phleboviruses, transmitted to humans by the bite of phlebotomine sandflies [15], belong to two distinct serocomplexes: the SFNV serocomplex including TOSV and the related Naples virus, and the SFSV serocomplex including Sicilian virus and the related Cyprus virus. Several of these are recognised human pathogens [16]. SFSV and SFNV are responsible for a self-resolving febrile illness called sandfly or phlebotomus fever [17,18]. SFCV, a variant of the SFSV, causes an acute febrile disease [19,20], whereas TOSV displays a neurotropism distinct from other phleboviruses. It is the only sandfly-borne virus to have been unambiguously associated with infection of the central nervous system and represents a major cause of meningitis and meningoencephalitis [21]. In endemic regions, such as countries in the Mediterranean basin, sandfly-borne viruses are especially common during the summer months, correlating with the life cycle of the vectors that transmit these viruses as revealed by serosurveys.

CCHFV is predominantly transmitted by the bite of an ixodid tick, causing a widespread viral infection that can be fatal in human beings [22]. The geographic range of CCHFV is the most extensive among the medically important tick-borne viruses [23], and has been reported in many countries including Africa, Asia, and the Middle East. The infection can develop into a severe hemorrhagic fever with a case fatality rate ranging from 5 to 30% [24,25]. Humans can also be infected through direct contact with infected patients during the acute phase of infection, or with blood or tissues from viraemic livestock [26].

Person-to-person transmission through blood transfusion and organ transplantation has been reported for some arboviruses [27]. Little is known, however, about the seroprevalence of these viruses in North Africa, and especially in Tunisia where human studies are limited to TOSV. Previous studies in Tunisia have revealed that 10% of patients with meningeal syndrome had IgM specific against TOSV [28], and a serological survey reported that 9.5% of healthy people were IgG positive against TOSV, suggesting that the diagnosis of TOSV infection is often neglected [29,30]. TOSV should be tested for in patients with meningitis and unexplained fever in Tunisia [31]. CCHFV has already been isolated from migratory birds in Morocco, whose migration path covers Tunisia [32]; therefore, Tunisia may also be at risk from CCHFV.

The aim of this study is to determine the potential seroprevalence of six viruses from two families of the order *Bunyavirales* among blood donors, in order to evaluate the activity of these viruses in the general population of central Tunisia.

## 2. Materials and Methods

### 2.1. Ethical Statement

This study was approved by the ethics committee of the University Hospital Farhat Hached, Sousse-Tunisia, with a protocol reference date: 1 April 2017.

### 2.2. Population Tested

Serum samples were collected from 377 blood donors between 1 August and 15 October 2017. Sera samples were stored for up to 12 months at −20 °C until required for analysis. The blood donors were recruited from the blood bank of the University Hospital Farhat Hached, Sousse, Tunisia. Adult blood donors who were asked to participate in this survey provided signed consent, and each participant filled out a fact sheet to reveal the presence of risk factors for arthropod-borne infections.

### 2.3. Serological Tests

Sera from blood donors were tested for the presence of immunoglobulin G (IgG) against each of the following viruses using commercial indirect immunofluorescence tests (IIFT Euroimmun, Lübeck, Germany): anti-Rift Valley fever virus (FI280a-1005 G and FI280a-1005 M); Crimean–Congo hemorrhagic fever virus mosaic 2 (FI279a-1005-2 G and FI279a-1005-2 M); and sandfly fever mosaic virus (FI277a-1005-1 G et FI277a-1005-1 M), the latter enabling the simultaneous detection of four viral serotypes (TOSV, SFNV, SFSV, and SFCV). These kits were used according to the manufacturer’s instruction. The presence of IgM, determined in samples only after the positive identification of IgG, was detected via IIFT. Samples were diluted 1:100 in sample buffer and interpreted using fluorescence microscopy. For IgM detection, IgG was removed from patient sera via immunoabsorption.

### 2.4. Molecular Tests

Positive sera were transferred at less than −20 °C to Porton Down, UK, for molecular analysis. Viral RNA was extracted using the Qiamp viral RNA mini Kit (Qiagen, Hilden, Germany), and subjected to PCR assays for pan-flaviviruses [33,34], pan-phleboviruses [35,36], RVFV [37], and CCHFV [38].

### 2.5. Statistical Analyses

Statistical significance among the groups was determined using a Pearson chi-square test. SPSS version 11 was used to calculate the Chi2 and a *p* value of less than 0.05 was considered statistically significant.

## 3. Results

Among the 377 subjects enrolled in this study, 308 (81.7%) were male and 69 (18.3%) female. The average age was 36.39 ± 9.82 years with extremes ranging from 19 to 60 years. The blood donors had many different origins and were residents of various cities in central Tunisia, the predominant cities being Sousse (67.9%), Monastir (16.4%), and Kairouan (5.6%). The place of residence was rural in 31.3%, semi-rural in 40.8%, and urban in 27.9% of cases. Of the participants, 80.6% had domestic or farm animals in the surrounding vicinity, and 60.5% recorded the presence of stagnant water (*p* = 0.09) within the vicinity of their living quarters.

A seropositivity of 17.3% (65/377) was recorded for at least one of the phleboviruses tested from the *Phenuviridae* family, while only one sample (0.3%) was IgG positive for the *Nairoviridae* family (CCHFV). Antibodies against TOSV were the most frequently detected (13.3%), followed by SFCV (2.9%), RVFV (1.9%), SFSV (1.3%), and SFNV (1.1%) (Table 1).

Of 377 donors, 60 (15.9%) were IgG positive for sandfly-borne phlebovirus, showing reactivity against at least one of the viruses tested in the indirect immunofluorescence assay. Samples showing single specific reactivity were 43 (11.4%) for TOSV, 5 (1.3%) for SFCV, and 1 (0.3%) for both SFSV and SFNV (Table 1). The dual reactivities observed in the IIFT were for SFSV + SFCV (3 cases), TOSV + SFNV (2 cases), and TOSV + SFCV (2 cases). However, one case was positive for all four sandfly-borne phleboviruses tested.

Positive sera were assessed for the presence of viral nucleic acid via multiple PCR assays, including two pan-viral assays for each of flaviviruses and phleboviruses and then virus-specific assays for RVFV and CCHFV. All PCR results were negative.

The seroprevalences varied according to the place of residence. Thus, the percent IgG positive for at least one of the tested viruses from serum collected in each governorate was higher among those from Kairouan 33.4% (7/21), followed by Monastir 24.2% (15/62), Sousse 14.9% (38/256), and 13.2% (5/38) for the other governorates. No IgM positives were detected for any of the IgG positive samples and viral RNA was not detected via RT-PCR.

Of the 65 donors IgG positive for phleboviruses, 50.8% resided in rural areas, compared to 12.3% in urban areas (*p* < 0.001). Sixty of the positive donors recorded having animals in their living quarters (*p* = 0.009). Stagnant water was present in the vicinity of 46 of the participants but was absent in 19 cases (70.8% vs. 29.2%, *p* = 0.062). The seroprevalence of phlebovirus IgG was significantly higher amongst participants reporting chronic illness (7.7%) with a *p* value of 0.039. These chronic conditions were diabetes, hypertension, hypothyroidism, allergy, and rheumatism (one case each). No significant association was observed with age, sex, or history of travel (Table 2).

From the 60 sandfly-borne phlebovirus IgG positive donors, 56 (93.3%) recorded having animals present in their living quarters (*p* = 0.007), and 42 (70%) having stagnant water (*p* = 0.1) in the surrounding vicinity. The seroprevalence of sandfly virus IgG was significantly higher among participants residing in villages or rural areas (50%), compared to 11.7% in those inhabiting urban areas (*p* < 0.001). These risk factors were found to be significant when at least one of the tested phleboviruses was IgG positive (Table 2). Due to the lower number of positive samples for Cyprus and RVFV (n = 11 and n = 7, respectively), the significance threshold to ascertain risk factors was not reached.

## 4. Discussion

To our knowledge, this is the first sero-epidemiology survey among blood donors testing for human exposure to six viruses from the order *Bunyavirales* in the general population of central Tunisia. Moreover, dual reactivity was observed in nine cases: SFSV + SFCV in three cases (0.8%), and two cases (0.5%) for each of TOSV + SFNV, TOSV + SFCV, and TOSV + RVFV.

Recent studies have reported that phleboviruses are not geographically restricted to southern Europe, but are also present in North Africa [16]. Among the 377 donors enrolled in this study, a seroprevalence of 13.3% for TOSV, 2.9% for SFCV, 1.9% for RVFV, 1.3% for SFSV, and 1.1% for SFNV was determined for viruses tested in the phlebovirus genus, while the rate of CCHFV was about 0.3%. A total of 17.3% were IgG positive for at least one of the phleboviruses tested. Of these, 50.8% resided in rural areas. This seroprevalence was significantly higher compared to the 12.3% of donors who lived in urban areas (*p* < 0.001). In addition, 60 positive donors recorded the presence of animals in their living quarters (*p* = 0.009), and 46 positive donors lived in the vicinity of stagnant water (*p* = 0.062). These cases may be due to exposure to infected animals and wildlife in the rural area, and the uncontrolled movement of live animals and animal products, which is more likely to occur in rural areas where phleboviruses circulate and are adapted to arthropod transmission. Moreover, the IgG positivity against phleboviruses was significantly higher among participants with chronic disease (7.7%) with a *p* value of 0.039; however, due to the small number of individuals in this group (n = 5), with each having a different condition, there were insufficient data to define a specific correlation. No significant association of IgG reactivity with other risk factors (age, sex, or history of travel) was observed [39].

Focusing on the Tunisian central governorates, frequent exposure to phleboviruses in blood donors was revealed in this study, with regional variations in seroprevalence between governorates. Our results show that the percentage of IgG positivity for at least one of the phleboviruses tested was highest among donors from Kairouan (33.4%), followed by Monastir (24.2%) and Sousse (14.9%). Exposure to sandfly-borne phleboviruses other than TOSV was not identified in the Kairouan governorate, but was identified in Sousse and Monastir, with eight and two cases, respectively. Evidence for the co-circulation of sandfly viruses in addition to TOSV has been observed in many countries surrounding the Mediterranean [21,40]. These results demonstrate that sandfly-borne viruses are present in central Tunisia and must be considered in cases/outbreaks of febrile disease of unknown etiology [41]. It remains to be determined which strains of TOSV or closely related variants are in circulation.

Sandfly-borne phleboviruses are present in North Africa in regions where *Leishmania infantum*, the causative agent of zoonotic visceral leishmaniasis in the western Mediterranean basin, is presently affecting both dogs and humans [16,30]. Leishmania parasites are transmitted to humans by phlebotomine flies [42]. Recent studies have shown that sandfly-borne phleboviruses, namely TOSV and Punique virus, are present in Tunisia. These two viruses were detected and isolated from *Phlebotomus pernicious* and *Phlebotomus longicuspis*, which are considered possible vectors of phleboviruses. Both sandfly species are also the main vectors of *Leishmania infantum* where the governorate of Kairouan has endemic foci [16,43]. In addition, a high prevalence of TOSV antibodies (up to 40%) was reported among the Tunisian population living in endemic foci for leishmaniasis [18,31,44]. Dogs are the main reservoir hosts for *Leishmania infantum* and therefore are used as sentinels to assess the risk of zoonotic visceral leishmaniasis and other zoonotic vector-borne diseases. *Phlebotomus pernicious* and *Phlebotomus longicuspis* are widely distributed in Tunisia and, subsequently, we hypothesize a large distribution of sandfly-borne phleboviruses that may not be currently being diagnosed.

The Mediterranean countries have been identified as endemic regions for many sandfly-borne phleboviruses, especially TOSV [21,45]. Sandfly virus studies in Tunisia were often limited to TOSV, which is not only confined to southern Europe and the Middle East; there is a lot of evidence for their presence in North African countries such as Malta and Morocco, where the insect vectors are present [40,46]. Our results showed that 13.3% of healthy people have IgG anti-TOSV antibodies. This rate is roughly similar to that reported in blood donors from the south of France (12%) [47], but higher than that observed in Portugal (3.1% in patients without neurological signs) and lower than that observed in hyper-endemic areas such as in Italy, where a study revealed a seroprevalence of 77.2% in forestry workers in the Tuscany region [48]. Indeed, TOSV has been recognized as among the most important etiologic agents of meningitis and meningoencephalitis in endemic Mediterranean countries with varying seroprevalences, with 35% and 60% in Greece from populations living on the Ionian Islands and western mainland, respectively [49], 25% in Spain, and 20% in Cyprus [47,49].

Previous studies have shown that TOSV seroprevalence among healthy people in Tunisia varied from 12% in 2007 [47] to 9.5% in 2013 [29]. According to the serological survey of Sakhira et al., TOSV seroprevalence in Tunisia varied from 17.2% to 59.4% depending on the region in different bioclimatic zones, and seroprevalence rates of 7.5% and 38.1% for Toscana and Sicilian viruses, respectively, were found in the governorate of Kairouan [16,30]. Furthermore, Toscana infection was ascribed responsibility for approximately 10% of cases of neurological disease in Tunisia [28,50]. The higher rate of TOSV observed in the Kairouan governorate can be explained by the exposure of people in this region to certain risk factors associated with TOSV infection, such as residing in rural areas, the presence of animals and/or stagnant water, working outdoors, and the frequent sightings of mosquitoes/sandflies [21]. During the hot season, populations living in these areas are likely to spend more time outdoors and therefore are at greater risk of being bitten by sandflies. Geographic and climatic conditions (temperature and humidity), factors that affect vector distribution and abundance, could explain the regional variations in seroprevalence between different governorates of Tunisia. The higher temperatures in some governorates may also affect the ability of vectors to efficiently transmit the virus in the field [51], and environmental changes—mainly due to intense irrigation in arid areas of central Tunisia—may also impact the emergence of TOSV and other sandfly-borne phleboviruses. In northern Nigeria, similar ecological factors such, as rain-fed crops, have been associated with RVFV in similar semi-arid and arid areas [52]. Thus, the establishment of a national surveillance sentinel network, anti-vector interventions, and a screening strategy—such as the seasonal screening of blood donors for phleboviruses correlating with the life cycle of vectors linked to transmitting these viruses—should be implemented [30].

A meta-analysis conducted to evaluate the association between various possible risk factors and RVF infection concluded that being male, increased age, contact with various types of animals, birthing or skinning animals, and eating raw animal products—including meat and unpasteurized milk which are common practices among community members living in rural area [12]—are statistically significant risk factors for RVF infection. However, this association was not found in this study, probably due to the low percentage of RVFV IgG seropositives [39].

CCHF is the most widespread tick-borne disease in the world [22] and has been reported in more than 30 countries including Africa, Asia, and the Middle East [24]. CCHFV has a transmission cycle involving tick and vertebrate hosts and is transmitted by the bite of ixodid ticks [25]. It has been isolated from several species of Ixodidae collected from migratory birds in Morocco that have a geographical distribution that also covers Tunisia. In our study, a seroprevalence of 0.03% was observed amongst healthy blood donors. This rate is lower than that reported in other endemic countries, including Mauritania (7%), Greece (4.2%), Kosovo (4%), Bulgaria (2.8%), Turkey (2.3%), and Iran (12%) [32].

The serological data obtained in this study confirm the circulation of TOSV and other bunyaviruses in central Tunisia. Only markers of past viral infections were found in our study with no samples positive for IgM antibodies. Alongside a negative result for RNA analysis—unsurprising given the only results were IgG—suggests the absence of an epidemic during the study period. However, a lack of sensitivity in relation to either the techniques used or to the brief window of viremia cannot be excluded [50,51].

Some other limitations of this study need to be mentioned: seroneutralization or confirmatory ELISA tests were not performed, hence the results must be interpreted carefully, as it is known that there is some antigenic cross-reactivity between phleboviruses, particularly amongst those belonging to the same serocomplex [47]. Commercial assays with appropriate controls were used for all sample testing, enabling confidence in the results, but unfortunately surplus material for confirmatory testing was not available as part of this specific study.

The fact that several arthropod-borne viruses have been isolated from blood donors on many occasions suggests the existence of a potential risk for transmitting these viruses through blood transfusion or organ transplantation [51]. Although no link with human disease is yet established after blood transfusion, the presence of phleboviruses antibodies in human sera raises questions about its possible pathogenicity and indicates a need for further investigation to better assess the prevalence of arboviruses in populations. Although attention is given to infectious diseases such as HIV during blood screening, our results indicate that there is a need for health authorities to consider the screening of arboviruses before blood transfusion. In addition, further work on the prevalence of arboviruses in Tunisia is warranted, including One Health collaborative efforts due to being zoonotic pathogens. Given the risk factors identified, future sample strategies may be more targeted to specific populations that are most likely to be exposed, especially those living in proximity to animals, the presence of stagnant water, and rural or peri-rural dwellings. In addition to studying responses in humans, expansion of work to animals and the arthropod vectors should also be considered.

## Figures and Tables

**Table 1:** IIFA sample results of phleboviruses detected (single, dual, or more reactive antibodies of tested viruses).

Viruses Tested from *Phenuiviridae* Family (n = 377)						
Phlebovirus Genus		IgG IIFT Positive n (%)				IgG Negative n (%)
		Single	Dual	All	Total	
Sandfly-borne phleboviruses	Toscana virus	43 (11.4%)	6 (1.6%)	1 (0.3%)	50 (13.3%)	327 (86.7%)
	Sicilian virus	1 (0.3%)	3 (0.8%)	1 (0.3%)	5 (1.3%)	372 (98.7%)
	Naples virus	1 (0.3%)	2 (0.5%)	1 (0.3%)	4 (1.1%)	373 (98.9%)
	Cyprus virus	5 (1.3%)	5 (1.3%)	1 (0.3%)	11 (2.9%)	366 (97.1%)
Total sandfly-borne phleboviruses		50 (13.3%)	9 (2.4%) *	1 (0.3%) *	60 (15.9%) *	317 (84.1%)
Rift Valley fever phlebovirus	Rift Valley fever virus	5 (1.3%)	2 (0.5%)	0	7 (1.9%)	370 (98.1%)
Total phleboviruses n (%)		55 (14.6%)	9 (2.4%) *	1 (0.3%) *	65 (17.3%) *	312 (82.7%)

* Dual or more cross-reactions are counted only once.

**Table 2:** Distribution of positive cases according to demographics and risk factors.

Blood Donors		Phleboviruses (n = 65)			Sandfly Viruses (n = 60)			Toscana Virus (n = 50)			Cyprus Virus (n = 11)			Rift Valley Virus (n = 7)		
		n	%	*p*-Value	n	%	*p*-Value	n	%	*p*-Value	n	%	*p*-Value	n	%	*p*-Value
Sex	Male	54	83.1	0.752	51	85.0	0.471	43	86.0	0.398	8	72.7	0.326	4	57.1	0.118
	Female	11	16.9		9	15.0		7	14.0		3	27.3		3	42.9	
Presence of animals	Yes	60	92.3	**0.009**	56	93.3	**0.007**	47	94.0	**0.010**	10	90.9	0.337	6	85.7	0.594
	No	5	7.7		4	6.7		3	6.0		1	9.1		1	14.3	
Presence of stagnant water	Yes	46	70.8	0.062	42	70.0	0.100	35	70.0	0.139	8	72.7	0.305	5	71.4	0.429
	No	19	29.2		18	30.0		15	30.0		3	27.3		2	28.6	
Localization	Centre	53	81.5	0.537	48	80.0	0.346	38	76.0	0.093	11	100	0.144	7	100	0.294
	Other	12	18.5		12	20.0		12	24.0		0	0.0		0	0.0	
Area	Rural	33	50.8	**0.001**	30	50.0	**0.001**	30	60.0	**0.001**	3	27.3	0.555	4	57.1	0.398
	Semi-rural	24	36.9		23	38.3		15	30.0		6	54.5		1	14.3	
	Urban	8	12.3		7	11.7		5	10.0		2	18.2		2	28.6	
Stay in another governorate	Yes	40	61.5	0.923	37	61.7	0.909	32	64.0	0.641	5	45.5	0.222	3	42.9	0.269
	No	25	38.5		23	38.3		18	36.0		6	54.5		4	57.1	
Record of travel	Yes	7	10.8	0.902	7	11.7	0.714	7	14.0	0.362	0	0.0	0.296	0	0.0	0.463
	No	58	89.2		53	88.3		43	86.0		11	100		7	100	
Chronic disease	Yes	5	7.7	**0.039**	5	8.3	**0.028**	5	10.0	**0.013**	0	0.0	0.697	0	0.0	0.796
	No	60	92.3		55	91.7		45	90.0		11	100		7	100	

Bold and underlined values indicate where significance threshold reached (*p* < 0.05).

## Data Availability

All data are presented within this manuscript.
